# A 20:1 synergetic mixture of cafedrine/theodrenaline accelerates particle transport velocity in murine tracheal epithelium via IP_3_ receptor-associated calcium release

**DOI:** 10.3389/fphar.2023.1155930

**Published:** 2023-08-16

**Authors:** Götz Schmidt, Gerrit Rienas, Sabrina Müller, Fabian Edinger, Michael Sander, Christian Koch, Michael Henrich

**Affiliations:** ^1^ Department of Anesthesiology, Operative Intensive Care Medicine and Pain Therapy, Justus Liebig University Giessen, Giessen, Germany; ^2^ Department of Anesthesiology, Intensive Care Medicine, Emergency Medicine, Vidia St. Vincentius-Clinic Karlsruhe gAG, Karlsruhe, Germany

**Keywords:** mucociliary clearance, ciliary beat frequency, Akrinor, perioperative, ciliary activity

## Abstract

**Background:** Mucociliary clearance is a pivotal physiological mechanism that protects the lung by ridding the lower airways of pollution and colonization by pathogens, thereby preventing infections. The fixed 20:1 combination of cafedrine and theodrenaline has been used to treat perioperative hypotension or hypotensive states due to emergency situations since the 1960s. Because mucociliary clearance is impaired during mechanical ventilation and critical illness, the present study aimed to evaluate the influence of cafedrine/theodrenaline on mucociliary clearance.

**Material and Methods:** The particle transport velocity (PTV) of murine trachea preparations was measured as a surrogate for mucociliary clearance under the influence of cafedrine/theodrenaline, cafedrine alone, and theodrenaline alone. Inhibitory substances were applied to elucidate relevant signal transduction cascades.

**Results:** All three applications of the combination of cafedrine/theodrenaline, cafedrine alone, or theodrenaline alone induced a sharp increase in PTV in a concentration-dependent manner with median effective concentrations of 0.46 µM (consisting of 9.6 µM cafedrine and 0.46 µM theodrenaline), 408 and 4 μM, respectively. The signal transduction cascades were similar for the effects of both cafedrine and theodrenaline at the murine respiratory epithelium. While PTV remained at its baseline value after non-selective inhibition of β-adrenergic receptors and selective inhibition of β_1_ receptors, cafedrine/theodrenaline, cafedrine alone, or theodrenaline alone increased PTV despite the inhibition of the protein kinase A. However, IP_3_ receptor activation was found to be the pivotal mechanism leading to the increase in murine PTV, which was abolished when IP_3_ receptors were inhibited. Depleting intracellular calcium stores with caffeine confirmed calcium as another crucial messenger altering the PTV after the application of cafedrine/theodrenaline.

**Discussion:** Cafedrine/theodrenaline, cafedrine alone, and theodrenaline alone exert their effects via IP_3_ receptor-associated calcium release that is ultimately triggered by β_1_-adrenergic receptor stimulation. Synergistic effects at the β_1_-adrenergic receptor are highly relevant to alter the PTV of the respiratory epithelium at clinically relevant concentrations. Further investigations are needed to assess the value of cafedrine/theodrenaline-mediated alterations in mucociliary function in clinical practice.

## Introduction

Mucociliary clearance is a pivotal physiological mechanism that protects the lung by ridding the airways of pollution, colonization by pathogens, and infection. Together with basal, suprabasal, and goblet cells, multiciliated epithelial cells constitute the complex mucociliary clearance function of the respiratory tract (Legendre et al., 2021). Herein, ciliary cells play a pivotal role in preventing the accumulation of debris and colonization by microbial pathogens via outward-directed transportation processes (Whitsett, 2018; Legendre et al., 2021). Cilia are located on the apical side of the respiratory epithelium along the airways, where they are constructed by unique structural proteins (Whitsett, 2018). Due to their continuous synchronized and orally directed motion, not only inhaled particles, but also complex compositions of mucus, electrolytes, and endogenous defensive substances can be removed from lower airways and can be coughed up under normal physiological conditions (Delmotte and Sanderson, 2006; Legendre, et al., 2021). Ciliary activity, which can be measured as ciliary beat frequency (CBF), or particle transport velocity (PTV) as its surrogate, is not only a complex process ensuring precise adaption to different physiological and pathophysiological settings, but its function is modified by many different endogenous and exogenous parameters (Delmotte and Sanderson, 2006; Legendre et al., 2021). Several known influencing factors accelerate PTV with a subsequent improvement in the mucociliary clearance. First, ciliary activity is modulated by the sympathetic and parasympathetic nervous systems. Their transmitters, (nor)adrenaline and acetylcholine, modulate second messengers, such as cyclic adenosine monophosphate (cAMP), cyclic guanosine monophosphate, and calcium, which induce alterations in CBF (Salathe, 2007; Wyatt, 2015). Second, ciliary cells are sensitive to local temperature, acid-base balance, humidity, mechanical stress, cytokines released during infection, and paracrine effects mediated by adjacent cells, like mast cells and macrophages (Sanderson and Dirksen, 1986; Kilgour et al., 2004; Sutto et al., 2004; Salathe, 2007; Weiterer et al., 2014; Perniss et al., 2020). Third, the formation of reactive oxygen species and pathogenic bacterial or fungal components mediates opposing effects and may lead to a reduction in CBF with consequent impaired mucociliary clearance (Burman and Martin, 1986; Weiterer et al., 2015). Furthermore, congenital impairment of the coordination of mucociliary clearance can result in severe diseases, like primary cilia dyskinesia, where recurrent infections occur due to ineffective clearance (Horani and Ferkol, 2021). Ciliary dysfunction can also be acquired in many pathophysiologic conditions. Mucociliary clearance is impaired in critically ill patients in intensive care units, and it is further impaired during mechanical ventilation, which is associated with increased bacterial colonization and pneumonia (Nakagawa et al., 2005; Bassi et al., 2008). Furthermore, mucociliary clearance is impaired during general anesthesia with mechanical ventilation following endotracheal intubation. The degree of impairment depends not only on the temperature of the inspired air and the respirator settings, but also on the drugs used perioperatively. For example, some intravenous anesthetics, volatile anesthetics, and perioperative antagonists like neostigmine and sugammadex, have been studied for their influence on mucociliary clearance (Forbes and Gamsu, 1979; Kesimci et al., 2008; Ozciftci et al., 2022). However, the impact of drugs used to treat perioperative hypotension, which is a frequently observed condition in clinical practice, remains unclear. In Germany, a fixed 20:1 combination of cafedrine and theodrenaline (Akrinor®, Ratiopharm GmbH, Ulm, Germany) has been the main drug used to treat intraoperative hypotension or hypotensive states due to emergency situations since the 1960s (Heller et al., 2015; Bein et al., 2017; Weitzel et al., 2018). Cafedrine is composed of covalently linked theophylline and norephedrine, and theodrenaline is formed from theophylline and noradrenaline in the same manner (Bein et al., 2017; Kloth et al., 2017). These compounds are administered as an intravenous bolus in adults, and they restore mean arterial blood pressure by increasing preload, cardiac stroke volume, and cardiac output (Bein et al., 2017; Eberhart et al., 2018; Weitzel et al., 2018). The clinical effects of cafedrine/theodrenaline are provided through β1-and α-adrenoreceptor stimulation, while nonspecific inhibition of phosphodiesterases is thought to enhance their response (Bein et al., 2017; Kloth et al., 2017). In contrast with synthetic vasopressors (e.g., ephedrine, phenylephrine), systemic vascular resistance and heart rate remain mostly unaffected, which makes cafedrine/theodrenaline especially appealing in obstetric surgery (Heller et al., 2015; Bein et al., 2017; Kranke et al., 2021). Although cafedrine/theodrenaline has been widely used for decades, little is known about its pharmacodynamics and pharmacokinetics in specific end organs (Heller et al., 2015; Bein et al., 2017). This is rather surprising, as the unique combination of three single drugs (theophylline, norephedrine, noradrenaline) in a 20:1 mixture can produce various effects *in vivo*, which have recently been evaluated in human atrial myocardium (Kloth et al., 2017). However, the influence of cafedrine/theodrenaline on mucociliary clearance has not yet been investigated. Consequently, the aim of our experiments was to evaluate the influence of cafedrine/theodrenaline on PTV, which indicates mucociliary clearance in the lower airways. Mouse tracheae were exposed to the clinically applied 20:1 mixture of cafedrine/theodrenaline and the individual substances alone. Our experiments aimed to evaluate the interaction between cafedrine and theodrenaline to assess the effects of the individual components and to reveal the signaling cascades provoking an alteration in PTV. Therefore, specific inhibitory substances were applied to elucidate signal transduction cascades in the epithelium and inside the epithelial cell itself. Further experiments were conducted to determine the value of intracellular calcium release, its origin, and the value of extracellular calcium entry.

## Materials and methods

### Tracheal preparation and imaging

**FIGURE 1:**
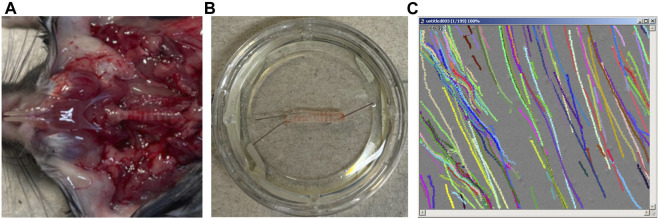
Tracheal preparation, microscopy, and particle tracking. **(A)** The trachea is dissected with a parasternal incision of the thorax and a median incision of the throat. **(B)** The trachea was fixed so that the pars membranacea, including the musculus trachealis, faced upward. **(C)** Particle tracks were recorded and processed offline.

Male C57BL6J mice weighing 25–35 g (aged 12–15 weeks) were purchased from Charles River (Sulzfeld, Germany). All procedures involving animals were conducted in compliance with the European legislation for the protection of animals used for scientific purposes and the standards for animal experiments according to the German animal welfare law. The experiments were approved by the local committee for animal care of the regional council (Permit number 813_M, regional council of Giessen, Germany). After deep isoflurane (Baxter, Unterschleissheim, Germany) narcosis, animals were sacrificed by cervical dislocation. The following steps were performed immediately within 30 min after euthanasia. The trachea was dissected with a parasternal incision of the thorax and a median incision of the throat (Figure 1A). The trachea was then gently disconnected by slicing cranial to its bifurcation and directly caudal to the larynx. The trachea was immediately transferred to a Delta T culture dish (Bioptechs, Butler, PA, USA) containing 2 mL of preheated 4-(2-hydroxyethyl)-1-piperazine ethanesulfonic acid (HEPES) buffer at pH 7.4 and 30°C. The dish was pre-coated with Sylgard polymer (Dow Corning, Wiesbaden, Germany) to allow precise positioning of the trachea with two minutiae (Fiebig Lehrmittel, Berlin, Germany). The trachea was fixed so that the cartilage arches faced the Sylgard polymer and the pars membranacea, including the musculus trachealis, faced upward. During the following fine preparations, connective tissues and surrounding blood vessels were gently resected using spring scissors (Vannas-Tübingen, FST, Heidelberg, Germany). Finally, preparation was completed when the musculus trachealis was cut open in a longitudinal direction and the respiratory epithelium was directly visualized (Figure 1B). After the HEPES buffer was replaced, the trachea was transferred to the stage holder of the upright transmission light microscope (BX50 WI, Olympus, Hamburg, Germany). A temperature control unit maintained a constant temperature of 30°C in the center of the buffer solution, where the trachea had been placed. According to the previously published methods, the optimal measuring conditions were realized at 30°C, although the PTV might be slightly slower than real time (Weiterer et al., 2015; Müller et al., 2021). Subsequently, 3 µL of polymer particles (Dynabeads, Dynal Biotech GmbH, Hamburg, Germany) with a mean diameter of 2.8–4.5 µm were added to the buffer solution. The tracheal epithelium was then focused between to cartilages in bright-field mode using a 20 × water immersion lens (BW50 WI, Olympus, Hamburg, Germany), allowing the measurement of PTV indicated by the controlled motion of the Dynabeads along the tracheal epithelium.

### Measurement of PTV

After a 30-min resting period, subsequent 80-min observation period started, where repeated measurements of PTV were performed under the influence of the different drugs. During the first 72 min of the observation period, measurement of PTV was conducted every 3 min. Afterward, adenosine triphosphate (ATP) was applied to confirm the viability of the tracheal epithelium leading to a maximal increase in PTV; measurements of PTV were then performed every 2 min to the end of the experiment at minute 80. At each timepoint, short movie sequences were recorded with high sampling rates using TiLLvisION Imaging software (Till Photonics, Gräfeling, Germany), as described previously (Weiterer et al., 2014). In brief, each video sequence consisted of 200 images taken during a period of 16.726 s (one image/83.63 m), where approximately 200–400 particle tracks were recorded (Supplementary Video S1). Subsequent offline processing was performed using Image Pro Plus analysis software (Media Cybernetics, Rockville, MD, United States). After background subtraction, the images were converted in grey scale, and the formerly dark Dynabeads appeared as bright images. In greyscale, the 12-bit film was reduced to an eight-bit film to realize the monitoring of individual particle pathways (Figure 1C). Particles with less than 15% lateral deviation were included in the analysis. From these measurements, the average PTV was calculated for each timepoint.

### Statistical analysis

The absolute PTV value was standardized to 100% after the resting time and prior to the observation period. Tracheal preparations and PTV measurements were only included in the statistical analyses when tracheal preparations showed directed particle motion during the whole observation period, and a clear response to the application of ATP was detected at the end of the experiments, immediately resulting in normalized PTV values of more than 200%. However, when high concentrations were applicated to obtain the concentration-response relationship, maximum PTV was already reached and could not be further accelerated by ATP. In this case, tracheal preparations were still included when directed particle motion was preserved during the whole observation period, and replicate experiments yielded similar results. Absolute values of basal PTV per experimental group prior to the standardization are shown in Supplementary Table S1. PTV values are presented as mean and standard error of the mean (SEM). Median effective concentrations (EC_50_) were calculated using the Hill equation. The Mann-Whitney U test was used to compare equivalent measurement points from the different experiments, while the Wilcoxon rank-sum test was used to compare paired variables. In general, two-tailed values of *p* < 0.05 were considered statistically significant, while issues with multiple comparisons were counteracted by adjusting the α level according to the Bonferroni correction.

### Drugs and buffer solutions

The historically established 20:1 ratio of cafedrine/theodrenaline is related to mass but not molarity; therefore, due to the different molar masses of cafedrine (357.41 g/mol) and theodrenaline (375.38 g/mol), a 1 M solution of cafedrine/theodrenaline 20:1 with a molar mass of 7,882.98 g/mol consists of 1 M theodrenaline, and approximately 21 M cafedrine. Preparations and experiments were performed in HEPES solution consisting of 10 mM HEPES, 5.6 mM KCl, 2.2 mM CaCl_2_, 11 mM glucose, 136 mM NaCl, and 2.2 mM MgCl_2_. NaOH was used to adjust the pH to 7.4 at 30°C. To realize the experiments in Ca^2+^-free solutions, CaCl_2_ was substituted with 1 mM ethylene glycol tetraacetic acid. The following drugs were applied during the experiments: 2-aminoethoxydiphenylborane (2-APB, 40 µM diluted in 8 µL dimethyl sulfoxide (DMSO), TOCRIS Bioscience, Bristol, United Kingdom), ATP (150 µM in 3 µL H_2_O, Sigma-Aldrich, St. Louis, MO, United States), cafedrine (408 µM diluted in 100 µL H_2_O, Arevipharma, Radebeul, Germany), cafedrine/theodrenaline 20:1 (0.47 µM diluted in 100 µL H_2_O, Akrinor^®^, Ratiopharm, Ulm, Germany), caffeine (30 mM in 2 mL HEPES, Roth, Karlsruhe, Germany), CGP20712A (100 µM diluted in 20 µL H_2_O, TOCRIS Bioscience, Bristol, United Kingdom), H-89 (10 µM diluted in 20 µL DMSO, Sigma-Aldrich, St. Louis, MO, United States), ICI-118,551 (100 µM diluted in 20 µL H_2_O, TOCRIS Bioscience, Bristol, UK), theodrenaline (4 µM diluted in 100 µL H_2_O, Arevipharma, Radebeul, Germany), and U-73122 (7.5 µM diluted in 4 µL DMSO, Enzo Life Sciences, Farmingdale, NY, United States). The reported drug concentrations were achieved during the experiments after applying the stock solution to the buffer solution in the recording chamber.

## Results

### Effects of cafedrine, theodrenaline and 20:1 cafedrine/theodrenaline

**FIGURE 2:**
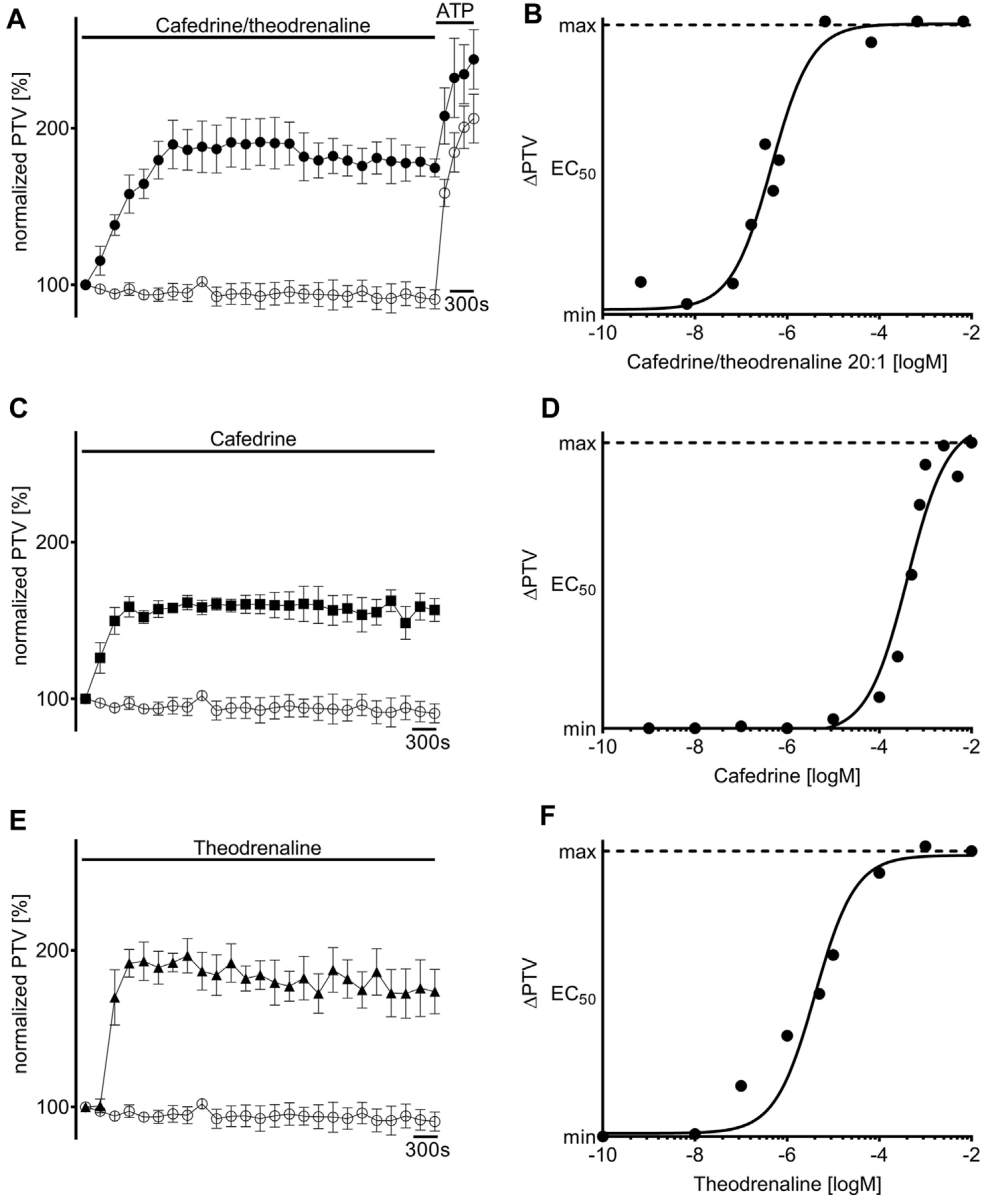
Particle transport velocity (PTV) is increased by cafedrine/theodrenaline, cafedrine alone and theodrenaline alone. Application of **(A)** 20:1 cafedrine/theodrenaline (●, 0.46 µM), **(C)** cafedrine alone (◼ 408 µM), and **(E)** theodrenaline alone (▲, 4 µM) provoked a steep and long-lasting increase in murine PTV, while the PTV remained constant around its baseline value in control experiments (○).Concentration-response relationship of **(B)** 20:1 cafedrine/theodrenaline, **(D)** cafedrine alone, and **(F)** theodrenaline alone, as described by the Hill equation. ⊥ standard error of the mean.

Under control conditions, murine PTV remained around baseline during the whole observation period (96% ± 2%). When cafedrine/theodrenaline, cafedrine alone, or theodrenaline alone were applied, PTV significantly increased almost immediately in all experiments (cafedrine/theodrenaline: 178% ± 12%, cafedrine: 160% ± 8%, theodrenaline: 196% ± 9%; each *p* < 0.001; Figures 2A,C,E). All three substances reached a sustained PTV plateau within a few minutes after application, which was largely maintained until the end of each experiment. The slowest increase lasted 18 min and was observed after the application of cafedrine/theodrenaline, while the most rapid increase was observed during the application of theodrenaline, reaching its plateau after a brief delay within 9 min. When ATP was applied at the end of the experiments, PTV was further accelerated, indicating the vitality of the tracheal epithelium; this is demonstrated for all the experiments by an example in Figure 2A. Supplementary Videos S1, S2 show raw movie sequences of baseline PTV and accelerated PTV following the application of cafedrine/theodrenaline 20:1. All three substances elevated the PTV in a concentration-dependent manner (Figures 2B,D,F), reaching maximum PTV values. At the highest concentration, PTV was not further increased by ATP, indicating full agonistic behavior of the three tested substances. The Hill equation fit the individual concentration-response curves and revealed EC_50_ values of 0.46 µM (3.6 μg/mL consisting of 3.42 μg/mL [9.6 µM] cafedrine and 0.17 μg/mL [0.46 µM] theodrenaline) for cafedrine/theodrenaline (Figure 2B), 408 µM (145.8 μg/mL) for cafedrine (Figure 2D), and 4 µM (1.5 μg/mL) for theodrenaline (Figure 2F). The following experiments aimed to evaluate the mediating effects and the individual value of cafedrine and theodrenaline when applied in a 20:1 combination. The clinical impact of different adrenoreceptors and the value of phosphodiesterases were initially studied because these target structures were known to be affected by theophylline, norephedrine, and noradrenaline.

### Extracellular signal transduction

**FIGURE 3:**
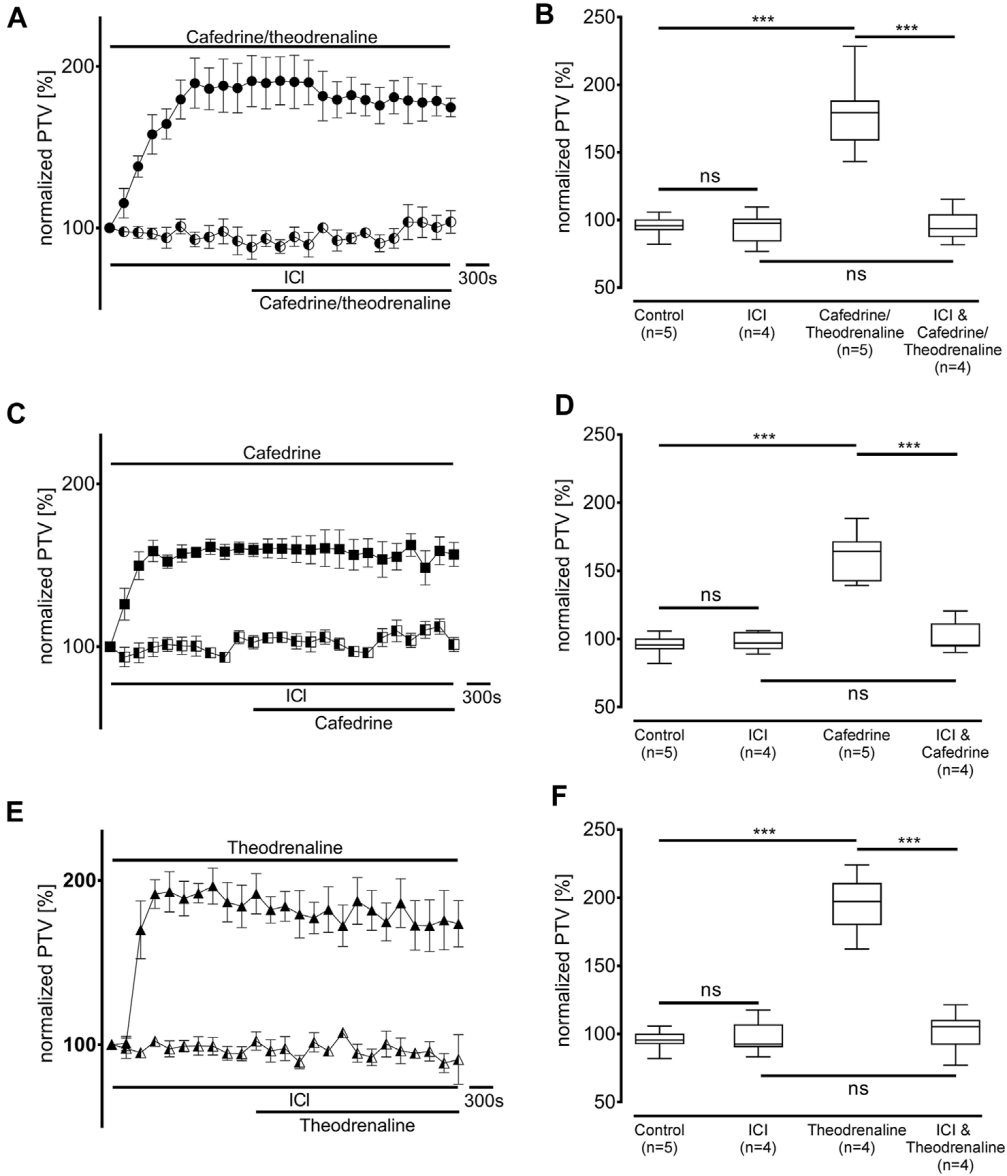
The increase in PTV depends on β-adrenergic receptor activation. Effects of **(A, B)** 20:1 cafedrine/theodrenaline, **(C, D)** cafedrine alone, and **(E, F)** theodrenaline alone completely vanished when β-adrenergic receptors were blocked by the non-selective inhibitor ICI. ICI alone did not provoke any alteration in the PTV. ***p < 0.001, ns: not significant. ● 20:1 cafedrine/theodrenaline, ◐ 20:1 cafedrine/theodrenaline + ICI 100 μM, ◼ cafedrine, ◧ cafedrine + ICI 100 μM, ▲ theodrenaline, ◭ theodrenaline + ICI 100 μM, ⊥ standard error of the mean, box and whisker plots indicate median, interquartile range (box), minimum and maximum (whiskers).

**FIGURE 4:**
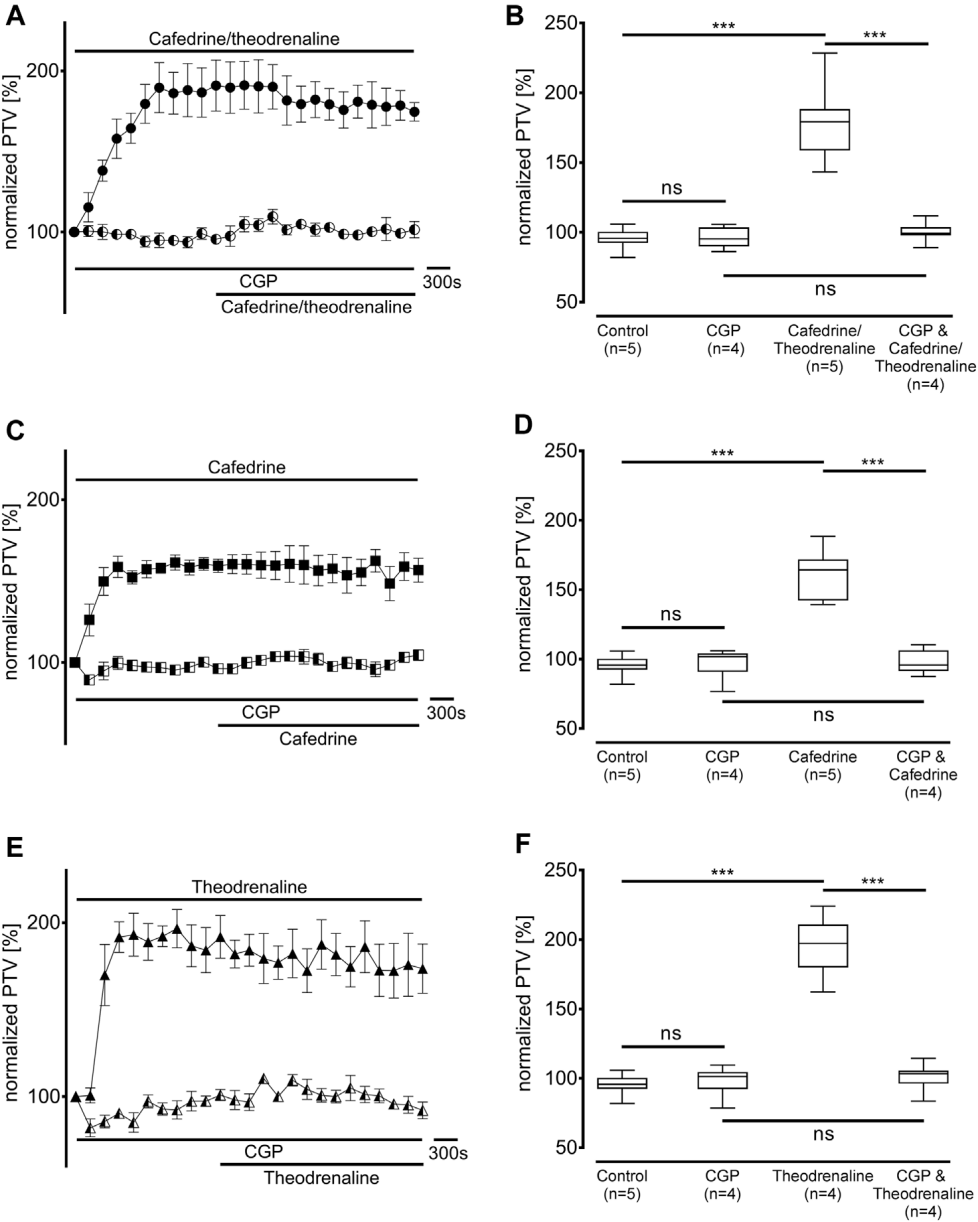
Alteration of PTV is completely dependent on β_1_-adrenergic receptor activation. When the selective β_1_-adrenergic receptor blocker CGP (100 µM) was applied, the effects of **(A, B)** 20:1 cafedrine/theodrenaline, **(C, D)** cafedrine alone, and **(E, F)** theodrenaline alone completely vanished. CGP alone did not provoke any alteration in the PTV. ***p < 0.001, ns: not significant. ● 20:1 cafedrine/theodrenaline, ◐ 20:1 cafedrine/theodrenaline + CGP 100 μM, ◼ cafedrine, ◧ cafedrine + CGP 100 μM, ▲ theodrenaline, ◭ theodrenaline + CGP 100 μM, ⊥ standard error of the mean, box and whisker plots indicate median, interquartile range (box), minimum and maximum (whiskers).

First, the non-selective blocker of β-adrenergic receptors ICI-118,551 (100 µM) was evaluated to determine the overall influence of β-adrenergic effects on the alteration in PTV. ICI-118,551 alone did not alter the baseline value of PTV, however, the increase in PTV was already completely blocked in the presence of ICI-118,551 when cafedrine/theodrenaline (95% ± 5%, *p* = 0.52, Figures 3A,B), cafedrine (100% ± 5%, *p* = 0.61, Figures 3C,D), or theodrenaline (102% ± 6%, *p* = 0.14, Figures 3E,F) were applied. To assess the individual value of the β_1_ receptor, the selective β_1_ receptor blocker CGP20712A was applied. Again, cafedrine/theodrenaline (95% ± 3%, *p* = 0.17, Figures 4A,B), cafedrine (98% ± 4%, *p* = 0.61, Figures 4C,D), and theodrenaline (99% ± 2%, *p* = 0.53, Figures 4E,F) were unable to provoke any alteration of the PTV compared to control conditions. Because a clinically relevant effect of other adrenergic receptors or the phosphodiesterases could not be assumed from these data, further experiments elucidating these mechanisms were waived.

### Intracellular signal transduction

**FIGURE 5:**
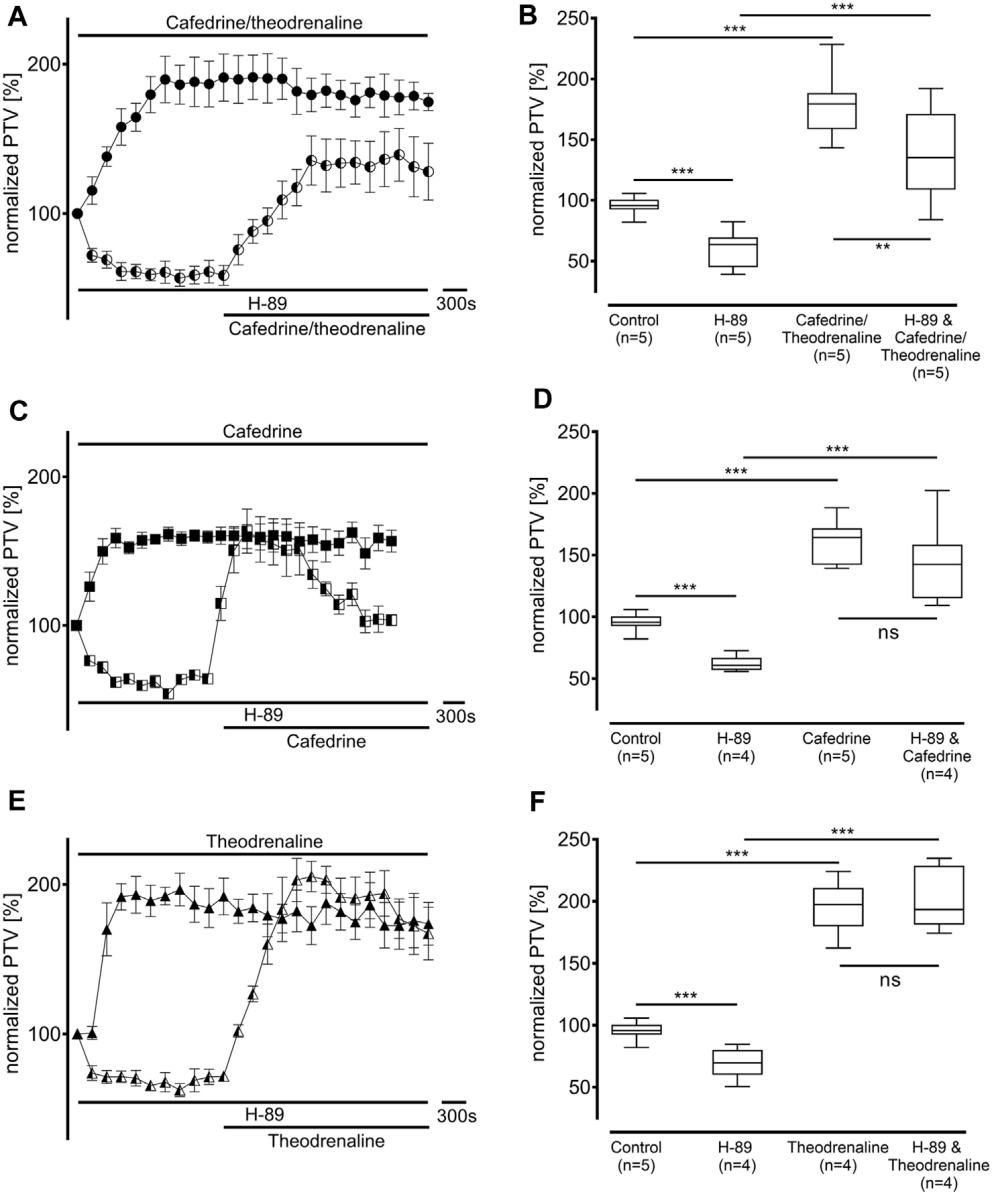
The increase in PTV was independent from protein kinase A (PKA). Baseline PTV decreased when the selective PKA inhibitor H-89 (10 µM) was applied. However, **(A, B)** 20:1 cafedrine/theodrenaline, **(C, D)** cafedrine alone, and **(E, F)** theodrenaline alone still provoked a steep increase in PTV. **p < 0.01, ***p < 0.001, ns: not significant. ● 20:1 cafedrine/theodrenaline, ◐ 20:1 cafedrine/theodrenaline + H-89 10 μM, ◼ cafedrine, ◧ cafedrine + H-89 10 μM, ▲ theodrenaline, ◭ theodrenaline + H-89 10 μM, ⊥ standard error of the mean, box and whisker plots indicate median, interquartile range (box), minimum and maximum (whiskers).

**FIGURE 6:**
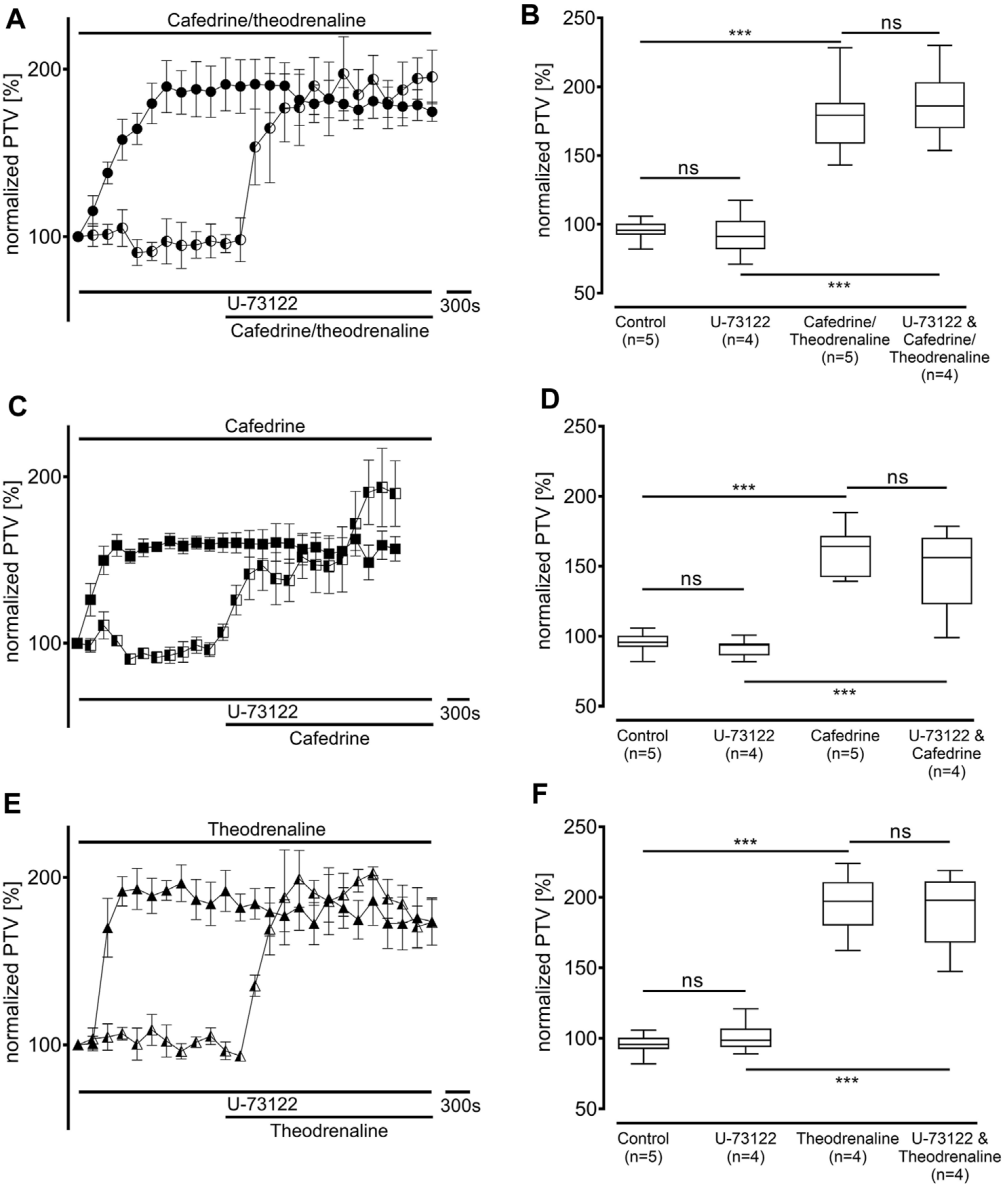
The increase in PTV was independent from phospholipase C (PLC). When the selective PLC inhibitor U-73122 (7.5 µM) was applied, **(A, B)** 20:1 cafedrine/theodrenaline, **(C, D)** cafedrine alone, and **(E, F)** theodrenaline alone still provoked a steep increase in PTV. ***p < 0.001, ns: not significant. ● 20:1 cafedrine/theodrenaline, ◐ 20:1 cafedrine/theodrenaline + U-73122 7.5 µM, ◼ cafedrine, ◧ cafedrine + U-73122 7.5 µM, ▲ theodrenaline, ◭ theodrenaline + U-73122 7.5 µM, ⊥ standard error of the mean, box and whisker plots indicate median, interquartile range (box), minimum and maximum (whiskers).

**FIGURE 7:**
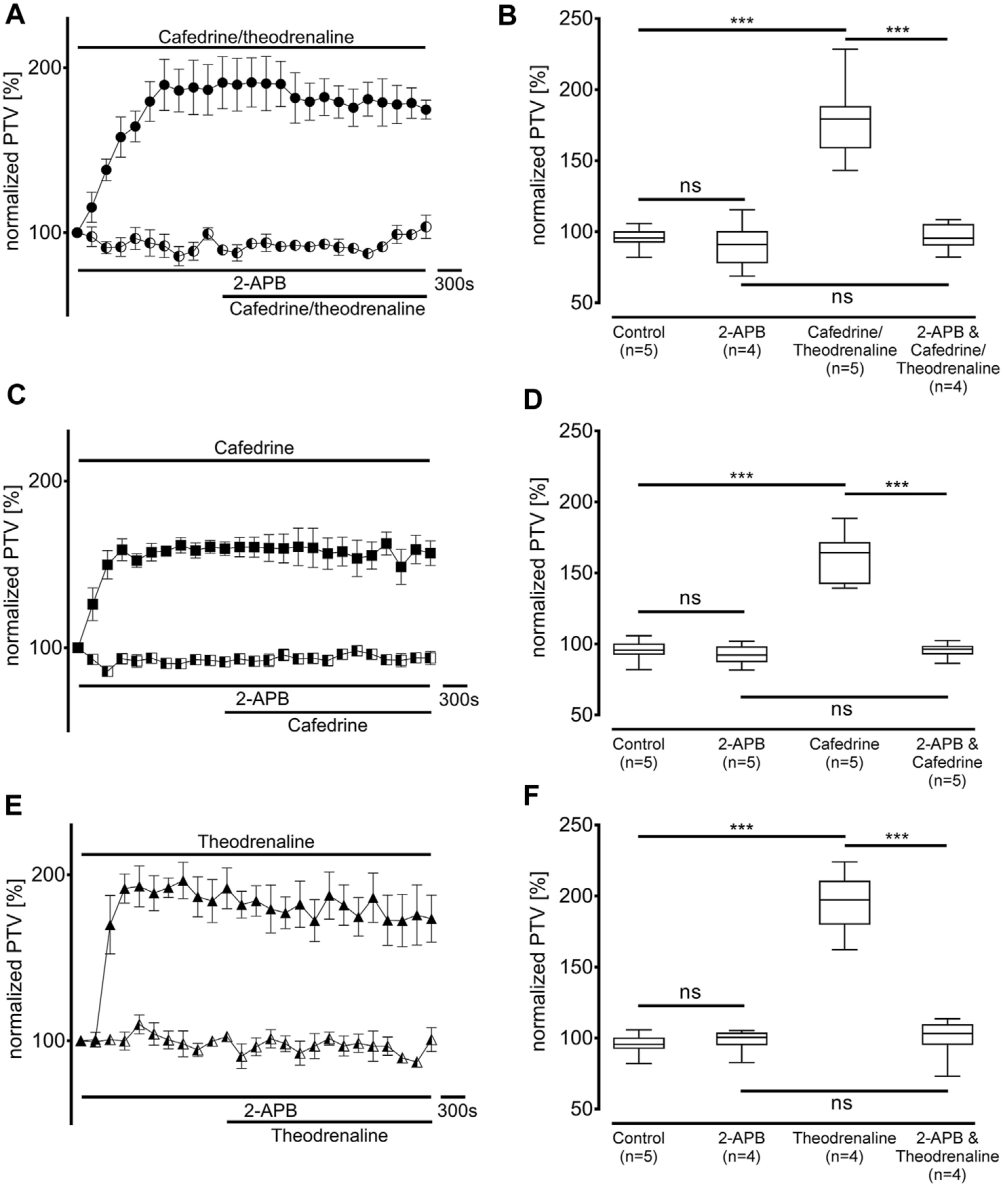
IP_3_ receptor activation was found to be the pivotal mechanism leading to the increase in PTV. IP_3_ receptors were selectively inhibited by 2-aminoethoxydiphenylborane (2-APB, 40 µM). When **(A, B)** 20:1 cafedrine/theodrenaline, **(C, D)** cafedrine alone, or **(E, F)** theodrenaline alone were subsequently applied, no increase in PTV was observed compared to control experiments. ***p < 0.001, ns: not significant. ● 20:1 cafedrine/theodrenaline, ◐ 20:1 cafedrine/theodrenaline + 2-APB 40 μM, ◼ cafedrine, ◧ cafedrine + 2-APB 40 μM, ▲ theodrenaline, ◭ theodrenaline + 2-APB 40 μM, ⊥ standard error of the mean, box and whisker plots indicate median, interquartile range (box), minimum and maximum (whiskers).

Since our experiments found the β_1_ pathway to be the pivotal mechanism provoking an alteration of PTV after the application of cafedrine/theodrenaline, further experiments were carried out on the cAMP-dependent protein kinase signal pathway. H-89 was used to inhibit the protein kinase A (PKA), which alone reduced basal PTV (59% ± 6%, *p* < 0.001 compared with control experiments, Figures 5B,D,F). However, cafedrine/theodrenaline, cafedrine alone, and theodrenaline alone were all able to induce a significant rise in PTV despite the presence of H-89 (each *p* < 0.001, Figure 5). Although the plateau value was slightly lower when cafedrine/theodrenaline was applied in the presence of H-89 (136% ± 18%, *p* = 0.004), cafedrine (145% ± 15%, *p* = 0.076) and theodrenaline (201% ± 11%, *p* = 0.755) alone produced comparable PTV values. When phospholipase C (PLC) was inhibited using U-73122 (7.5 µM), baseline value of PTV remained unchanged; however, PTV still increased following the administration of cafedrine/theodrenaline, cafedrine alone, and theodrenaline alone (each *p* < 0.001, Figure 6). PTV plateau values of cafedrine/theodrenaline (196% ± 12%, *p* = 0.236, Figures 6A,B), cafedrine (162% ± 19%, *p* = 0.420, Figures 6C,D), and theodrenaline (191% ± 13%, *p* = 0.755, Figures 6E,F) alone were all comparable to the PTV observed without PLC inhibition. To further evaluate alternative signal transduction cascades, IP_3_ receptors were inhibited using 2-APB. While 2-APB alone did not provoke any alteration in the baseline PTV, the increase previously seen after the application of cafedrine/theodrenaline (90% ± 7%, *p* = 0.943, Figures 7A,B), cafedrine alone (93% ± 3%, *p* = 0.831, Figures 7C,D), and theodrenaline alone (99% ± 2%, *p* = 0.093, Figures 7E,F) completely vanished. As these results indicated that intracellular calcium (Ca^2+^) release as crucial mechanism changing the PTV after the application of cafedrine/theodrenaline, further experiments were carried out on the value and the origin of intra- and extracellular Ca^2+^.

### Utilization of Ca^2+^ stores and Ca^2+^-dependent effects on PTV

**FIGURE 8:**
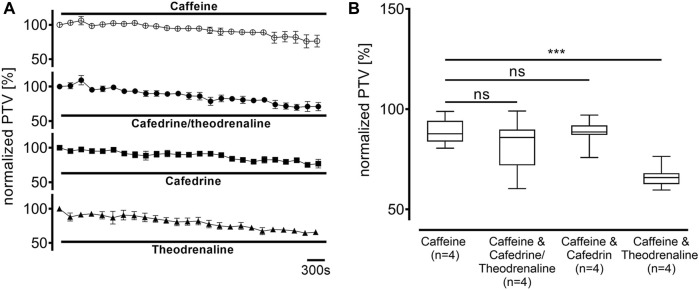
Intracellular Ca^2+^ stores were depleted by caffeine (30 mM), which subsequently decreased PTV. When **(A)** 20:1 cafedrine/theodrenaline, cafedrine alone, or theodrenaline alone were applied, a decrease from baseline was inexorable and **(B)** no increase was observed. ***p < 0.001, ns: not significant. ○ caffeine 30 mM, ● 20:1 cafedrine/theodrenaline + caffeine 30 mM, ◼ cafedrine + caffeine 30 mM, ▲ theodrenaline + caffeine 30 mM, ⊥ standard error of the mean, box and whisker plots indicate median, interquartile range (box), minimum and maximum (whiskers).

**FIGURE 9:**
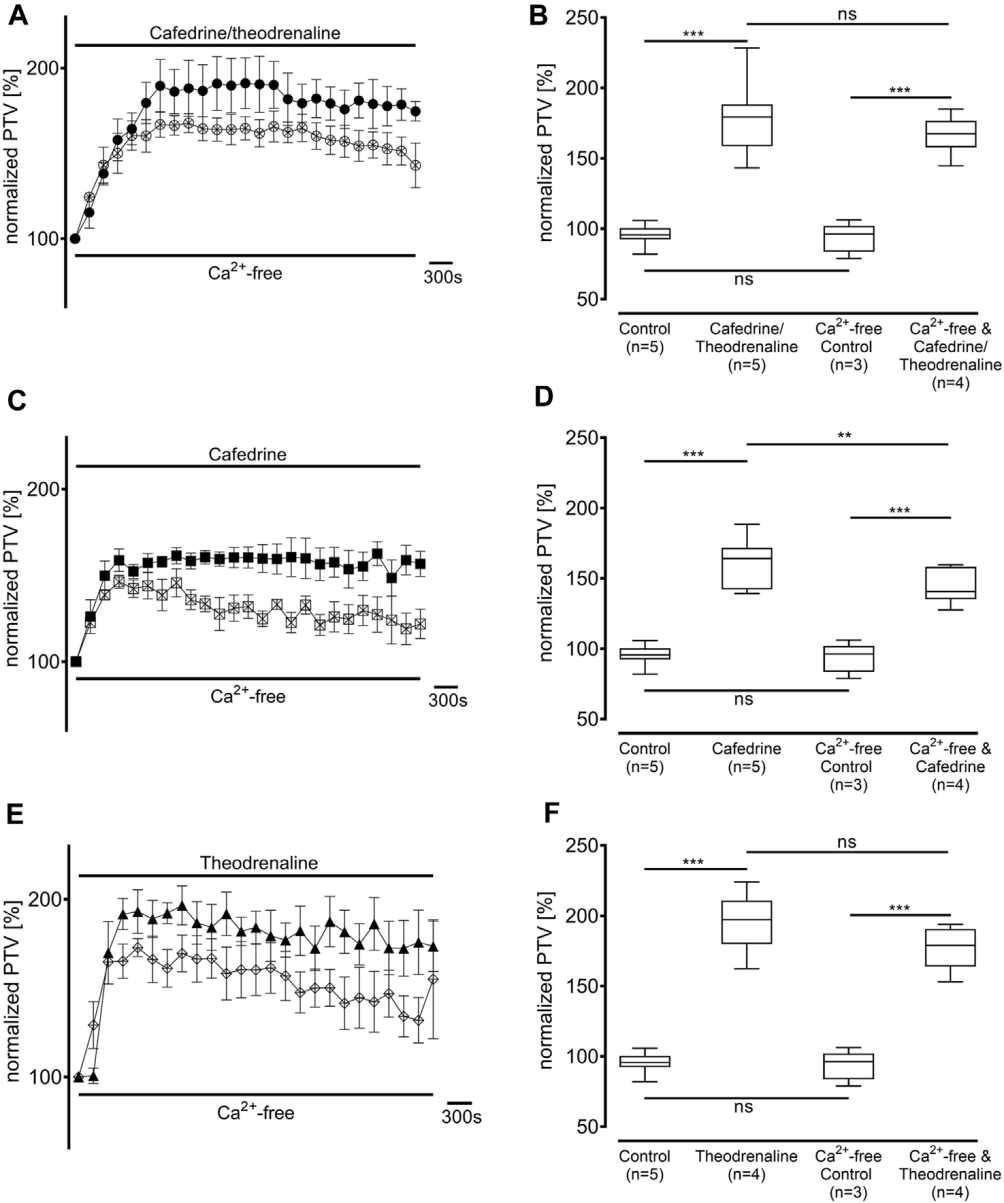
The PTV increase is largely independent from extracellular Ca^2+^ entry. PTV was measured in Ca^2+^-free buffer solution after **(A, B)** 20:1 cafedrine/theodrenaline and **(E, F)** theodrenaline alone were applied. However, PTV was significantly lower when PTV triggered by **(C, D)** cafedrine was compared to Ca^2+^-containing buffer solution. **p < 0.01, ***p < 0.001, ns: not significant. ● 20:1 cafedrine/theodrenaline, ○ 20:1 cafedrine/theodrenaline in Ca^2+^-free buffer, ◼ cafedrine, □ cafedrine in Ca^2+^-free buffer, ▲ theodrenaline, ◇ theodrenaline in Ca^2+^-free buffer, ⊥ standard error of the mean, box and whisker plots indicate median, interquartile range (box), minimum and maximum (whiskers).

Ca^2+^-free buffer solution was used to distinguish the origin of Ca^2+^ during the following experiments. PTV in Ca^2+^-free buffer solution did not differ from the Ca^2+^-containing control (94% ± 11%, *p* > 0.99). As most intracellular Ca^2+^ is stored in the endoplasmic reticulum, these stores were depleted by caffeine prior to the application of the three substances. Subsequently, a decline of the basal PTV was noted (89% ± 3%, *p* < 0.001); neither cafedrine/theodrenalin (83% ± 5%, *p* = 0.242) nor cafedrine (88% ± 2%, *p* = 0.843) or theodrenaline (66% ± 2%, *p* < 0.001 indicating further decline) alone were able to cause a significant rise in PTV compared with caffeine alone (Figure 8). Further experiments were conducted to assess the value of extracellular Ca^2+^ influx following treatment with cafedrine/theodrenaline (Figure 9). PTV increased equally after the application of cafedrine/theodrenaline (167% ± 6%, *p* = 0.251, Figures 9A,B), and theodrenaline alone (177% ± 7%, *p* = 0.021 did not reach the adjusted α level of 0.0125, Figures 9E,F); however, PTV without extracellular Ca^2+^ was slightly lower when cafedrine alone was applied (144% ± 6%, *p* = 0.005, Figures 9C,D). Furthermore, the plateau formed after the individual application of cafedrine and theodrenaline was less robust than that plateau observed with Ca^2+^-containing buffer solution (Figures 9A,C,E).

## Discussion

Our experiments revealed a significant increase in murine PTV, suggesting an alteration in mucociliary clearance, after the application of 20:1 cafedrine/theodrenaline, cafedrine alone, and theodrenaline alone. Existing clinical data reported a plasma concentration of 6 μg/mL after the intravenous application of one ampoule of 2 mL cafedrine/theodrenaline (Sternitzke et al., 1984). Therefore, our EC_50_ of 3.6 μg/mL represents clinically plausible concentrations in our experiments, as most applications of cafedrine/theodrenaline are given in 0.5–1 mL steps in daily routine practice. Therefore, *in vivo* effects of cafedrine/theodrenaline on mucociliary clearance are likely according to our experiments. Furthermore, it can be hypothesized that the transient intravasal and intraepithelial concentrations reached immediately after injection could be much higher than those observed after initial distribution to the body compartments. Our experiments were performed at a lower temperature compared with human body temperature; thus, clinical effects of cafedrine/theodrenaline on PTV might be even stronger. Interestingly, the EC_50_ of cafedrine was more than two log units higher than the EC_50_ of theodrenaline. Therefore, the initial alteration of the PTV in our experiments could largely be attributed to theodrenaline. However, theodrenaline alone cannot explain the concentration-response relationship of the 20:1 combination of cafedrine and theodrenaline, because its concentration-response curve was shifted further left by almost one log unit. However, compared with the concentration-response curve of cafedrine, the remarkably low amount of cafedrine alone cannot explain the sharp increase in PTV observed in combination with theodrenaline. Therefore, we conclude that cafedrine and theodrenaline may initially interact in a highly synergistic manner at the respiratory epithelium. However, the pharmacological relevance of cafedrine has already been questioned in Kloth et al.’s *in vitro* investigation of the human atrial myocardium (Kloth et al., 2017). While clinical data suggested that the immediate response to cafedrine/theodrenaline was attributed to theodrenaline, the cafedrine response was observed with a 20-min delay (Sternitzke et al., 1984; Kloth et al., 2017). Therefore, it was hypothesized that cafedrine could act as prodrug, and only its metabolites were responsible for its clinical effect (Kloth et al., 2017). In our data, we did not observe any changes during the observation period as the PTV reached a robust plateau. However, despite raising the question of whether potential cafedrine metabolites could have been formed during our experimental setup, we still observed an immediate effect after its application. Unfortunately, to date, there are no studies evaluating the specific pharmacodynamics and potential metabolites of cafedrine, so this hypothesis remains unanswered.

To elucidate the interaction between cafedrine and theodrenaline regarding signal transduction cascades, we initially inhibited β-adrenergic receptors because these have been known to provide the main effect in increasing blood pressure and cardiac inotropy (Usichenko et al., 2006; Heller et al., 2015; Bein et al., 2017; Kloth et al., 2017). Although cafedrine and theodrenaline have different drugs linked to theophylline, their effect of increasing the PTV was completely abolished after the non-selective inhibition of β-adrenergic receptors using ICI-118,551 in high concentrations (Hoffmann et al., 2004). Selective inhibition of β_1_ receptors did not change these results; therefore, we conclude that cafedrine and theodrenaline both produce the increase in PTV exclusively via the β_1_ receptor. This conclusion is in line with experiments conducted on human myocardium and coronary arteries (Usichenko et al., 2006; Kloth et al., 2017; Weitzel et al., 2018). However, effects on α-adrenergic receptors were found in coronary arteries and mammary arteries when β-adrenergic receptors were inhibited (Usichenko et al., 2006; Kloth et al., 2017). These effects were solely attributed to theodrenaline, however, through our experiments, we did not observe any clinically relevant effect on the α-adrenergic receptors of the tracheal epithelium. Interestingly, peripheral artery vasoconstriction following the administration of theodrenaline seemed to be abolished by cafedrine, as it may act as a partial agonist at the α1-adrenergic receptor (Bein et al., 2017). One could hypothesize that the concentration of theodrenaline alone was not high enough to provoke a significant effect in our experiments, as α-adrenergic receptors were already shown to exist on the tracheal epithelium (Weiterer et al., 2015). However, Weiterer et al. only detected the α1D subtype and subsequently stated that the increases in PTV observed in their study were independent from α-adrenergic receptor activation because the PTV could not be increased after the application of α-receptor agonists (Weiterer et al., 2015). Consequently, we did not observe any difference between theodrenaline alone and the combination of cafedrine/theodrenaline. However, we can hypothesize that the alteration in PTV might be reduced in patients who use β-blocking agents, because same inhibitory effects accompanied by a slower and less powerful increase in arterial blood pressure were observed in clinical practice (Heller et al., 2015).

In addition to the activation of adrenergic receptors, our experiments did not indicate that the theophylline in cafedrine and theodrenaline provoked any effect at the tracheal epithelium by inhibiting phosphodiesterases. Although some trials reported increased mucociliary clearance and CBF after treatment with theophylline, data remain controversial (Matthys and Kohler, 1980; Ziment, 1987; Konrad et al., 1994). In general, only high concentrations of theophylline are able to provoke relevant effects, while low concentrations do not alter mucociliary clearance (Matthys and Kohler, 1980; Ziment, 1987). Therefore, our results are not only in line with clinical data, but *in vitro* data showed that only high, clinically irrelevant, concentrations of cafedrine/theodrenaline can provoke significant inhibition of phosphodiesterase in the human myocardium (Kloth et al., 2017).

As the change in mucociliary clearance was only evident when β_1_ receptors were stimulated, the synergistic effects of cafedrine and theodrenaline are likely to be provided there. The suggested mechanisms for synergistic effects are an increase in the agonist’s affinity or efficacy, and both mechanism are possible at β-adrenergic receptors (Lau et al., 1995; Liapakis et al., 2004; Ahn et al., 2018; Wang et al., 2021). The number of functional groups on epinephrine increase the affinity to β-adrenergic receptors (Liapakis et al., 2004). This can explain the higher receptor affinity and lower EC_50_ calculated for theodrenaline compared with cafedrine, because the linked noradrenaline contains two catechol hydroxy groups, while norephedrine does not. However, neither noradrenaline nor norephedrine contain the methyl group bound to the amino group as epinephrine does, but both molecules do contain the carboxy-bound hydroxy group. Furthermore, cafedrine has another methyl group covalently bound to the upward carbon. On the one hand, it can be hypothesized that providing more functional groups through the application of both cafedrine and theodrenaline at the receptor allows for higher receptor efficacy to be realized; on the other hand, allosteric modulators also improve the β-receptor’s affinity to agonists, which could also be induced by cafedrine, or theodrenaline (Ahn et al., 2018; Wang et al., 2021). However, it is possible that the presence of the catechol hydroxy groups increases the receptor’s affinity to cafedrine. Although this question will be difficult to answer in further studies, other epinephrine-derived substances could be combined with cafedrine or theodrenaline to distinguish their individual effects.

When intracellular signal transduction cascades following β_1_-receptor activation were analyzed, we found that PKA was not responsible for the effects triggered by the application of cafedrine/theodrenaline. In other experiments, no direct effects of cAMP were shown on CBF when PKA was blocked (Braiman et al., 1998; Wyatt et al., 1998). Therefore, we concluded that the increase in PTV was independent from direct effects triggered by PKA. Acetylcholine was described as a main initiator of PKA, in line with the decrease in baseline PTV observed after the inhibition of PKA alone preventing these basal effects (Zagoory et al., 2002). However, our results elucidated IP_3_ receptor activation as involving an alternative signaling cascade, the inhibition of which totally abolished the increase in PTV. Therefore, we conclude that IP_3_ receptor activation, but not PKA activity, is the pivotal mechanism leading to the rise in murine PTV triggered by cafedrine/theodrenaline. IP_3_ releases Ca^2+^ from intracellular stores after it is separated from phosphatidylinositol 4,5-bisphosphate by PLC, which certainly is not the main effector protein of the β_1_ receptor. However, IP_3_ is known to influence CBF, and it has already been shown that mammalian cAMP and PKA signaling pathways can mobilize Ca^2+^ from intracellular stores (Braiman et al., 1998; Barrera et al., 2004; Kuremoto et al., 2018). One suggested mechanism is that PKA shifts the affinity of the IP_3_ receptor to IP_3_ and thereby decreases the dose of IP_3_ that is required to provoke significant Ca^2+^ release; but cAMP alone can also activate PLC pathways (Schmidt et al., 2001). Furthermore, not only the α subunit of G-coupled receptors can activate PLC pathways as it is observed at the α_1_-adrenergic receptor, βγ subunits of β-adrenergic receptors can also activate PLC pathways (Galaz-Montoya et al., 2017; Madukwe et al., 2018; Fisher et al., 2020). However, PTV still increased equally following the administration of cafedrine/theodrenaline, cafedrine alone, and theodrenalin alone, when PLC was inhibited by U-73122. Our experiments depleting caffeine-sensitive Ca^2+^ stores, which are mainly found in the endoplasmic reticulum, confirmed that the IP_3_-receptor associated increase in PTV is due to Ca^2+^ release from the endoplasmic reticulum. We further evaluated secondary Ca^2+^ influx from extracellular spaces that occurs after Ca^2+^ release from the endoplasmic reticulum. This mechanism is referred to as store-operated calcium entry (SOCE), and it is mediated by stromal-interacting molecules activating ORAI channels (Spinelli and Trebak, 2016; Nguyen et al., 2018). To evaluate whether SOCE influences the PTV increase triggered by cafedrine/noradrenaline, we measured PTV in a Ca^2+^-free buffer solution, thereby excluding extracellular Ca^2+^ influx. While the immediate increase in PTV was comparable among all substances in Ca^2+^-free buffer solution, only cafedrine produced a significantly reduced PTV amplitude. Our experiments in Ca^2+^-free buffer solution provided further evidence that the PTV peak is produced by theodrenaline to a larger extent than cafedrine because its amplitude was comparable to the peak observed when cafedrine/theodrenaline were both applied. Although significant differences regarding a slightly reduced PTV amplitude were detected following cafedrine application, we do not support the high relevance of SOCE after the application of cafedrine/theodrenaline because the clinical relevance of this finding might be questioned according to the measured PTV peak values, because comparable peaks were seen when both cafedrine and theodrenaline were applied in their clinically used mixture.

Several limitations of our study must be acknowledged. First, we used isolated murine tracheae; therefore, the physiological integrity of the respiratory tract was not completely preserved. Consequently, physiological drug administration via capillary vessels could not be replicated, and atypical entrance (e.g., from the apical or lateral side of the trachea) of our tested drugs was preserved. Second, pharmacodynamics and pharmacokinetics might differ between murine tracheae and human lower airways, including the concentration-response relationship. Third, we used small sample sizes in each group; however, clear effects were observed, and larger sample sizes were prohibited by animal welfare regulations. Although clinically administered concentrations were comparable to the concentrations used in our experiments, further studies on human tissues are necessary to confirm the results of our study, and the clinical relevance of the observed effects should be assessed *in vivo*.

In conclusion, our experiments showed a sustained elevation in murine PTV when cafedrine/theodrenaline, cafedrine alone, or theodrenaline alone were applied. Both substances produce their effects via β_1_-adrenergic receptor-associated Ca^2+^ release that is ultimately triggered through IP_3_ receptor activation. We obtained evidence that the alteration in murine PTV observed after 20:1 cafedrine/theodrenaline is foremost induced by theodrenaline; however, their synergistic effects at the β_1_-adrenergic receptor are highly relevant to accelerate the PTV of the respiratory epithelium. Because the pharmacokinetics of cafedrine/theodrenaline remain unknown, further experiments including human tracheal epithelium and clinical studies are needed to evaluate the value of cafedrine/theodrenaline in altering mucociliary function in clinical practice.

## Data Availability

The raw data supporting the conclusion of this article will be made available by the authors, without undue reservation.

## Abbreviations

2-APB, 2-Aminoethoxydiphenylborane; ATP, Adenosine triphosphate; Ca^2+^, calcium; cAMP, cyclic adenosine monophosphate; CBF, ciliary beat frequency; DMSO, dimethyl sulfoxide; EC_50,_ median effective concentration; HEPES, 4-(2-hydroxyethyl)-1-piperazine ethanesulfonic acid; PKA, protein kinase A; PLC, phospholipase C; PTV, particle transport velocity; SEM, standard error of the mean; SOCE, store-operated calcium entry.
