# Supplementary material for: A 20:1 synergetic mixture of cafedrine/theodrenaline accelerates particle transport velocity in murine tracheal epithelium via IP3 receptor-associated calcium release
Source: Front Pharmacol. 2023 Aug 16;14:1155930. doi: 10.3389/fphar.2023.1155930 (PMC10466409; doi:10.3389/fphar.2023.1155930)
Supplement: Supplementary file 2 [file Table1.DOCX]

Supplementary Material

A 20:1 synergetic mixture of cafedrine/theodrenaline accelerates particle transport velocity in murine tracheal epithelium via IP_3_ receptor-associated calcium release

Götz Schmidt^1^*. Gerrit Rienas^1^. Sabrina Müller^1^. Fabian Edinger^1^. Michael Sander^1^. Christian Koch^1.†^. Michael Henrich^2.†^

^1^Department of Anesthesiology. Operative Intensive Care Medicine and Pain Therapy. Justus Liebig University Giessen. Rudolf-Buchheim-Strasse 7. 35392 Giessen. Germany

^2^Department of Anesthesiology. Intensive Care Medicine. Emergency Medicine. Vidia St. Vincentius-Clinic Karlsruhe gAG. Karlsruhe. Germany

† These authors share senior authorship.

*** Correspondence:**Götz Schmidt

Justus Liebig University Giessen.

Rudolf-Buchheim-Strasse 7.

35392 Giessen.

Email:Goetz.F.Schmidt@chiru.med.uni-giessen.de

# Supplementary Table

**Supplementary Table 1.** Absolute values of basal particle transport velocity per group prior to the standardization to 100%.

| Group | Mean PTV [µm/s] | SEM |
| --- | --- | --- |
| **Figure 1** | | |
| Control | 50.2 | 4.6 |
| Cafedrine/Theodrenaline | 46.6 | 4.5 |
| Cafedrine | 42.2 | 3.5 |
| Theodrenaline | 57.1 | 4.4 |
| **Figure 2** | | |
| Cafedrine/Theodrenaline & ICI-118.551 | 36.0 | 2.1 |
| Cafedrine & ICI-118.551 | 52.2 | 8.1 |
| Theodrenaline & ICI-118.551 | 43.1 | 4.9 |
| **Figure 3** | | |
| Cafedrine/Theodrenaline & CGP20712A | 39.0 | 2.5 |
| Cafedrine & CGP20712A | 55.2 | 6.9 |
| Theodrenaline & CGP20712A | 48.3 | 3.7 |
| **Figure 4** | | |
| Cafedrine/Theodrenaline & H-89 | 40.5 | 2.6 |
| Cafedrine & H-89 | 45.3 | 4.4 |
| Theodrenaline & H-89 | 44.7 | 6.3 |
| **Figure 5** | | |
| Cafedrine /Theodrenaline & U-73122 | 42.9 | 4.0 |
| Cafedrine & U-73122 | 36.4 | 2.8 |
| Theodrenaline & U-73122 | 39.0 | 4.4 |

**Supplementary Table 1.** (continued)

| **Figure 6** | | |
| --- | --- | --- |
| Cafedrine/Theodrenaline & 2-APB | 43.9 | 5.0 |
| Cafedrin& 2-APB | 47.0 | 2.7 |
| Theodrenaline & 2-APB | 38.4 | 3.6 |
| **Figure 7** |  |  |
| Caffeine Control | 71.9 | 9.8 |
| Cafedrine/Theodrenaline & Caffeine | 59.5 | 5.0 |
| Cafedrine & Caffeine | 73.6 | 7.4 |
| Theodrenaline & Caffeine | 74.7 | 5.3 |
| **Figure 8** |  |  |
| Ca^2+^-free Control | 48.8 | 3.4 |
| Ca^2+^-free Cafedrine | 45.7 | 4.1 |
| Ca^2+^-free Cafedrine/Theodrenaline | 54.2 | 9.1 |
| Ca^2+^-free Theodrenaline | 41.1 | 2.8 |
| PTV: particle transport velocity; SEM: Standard error of the mean | | |

## Supplementary Videos

**Supplementary Video 1.** Synchronized and orally directed particle motion in murine trachea during resting conditions.

**Supplementary Video 2.** Accelerated particle transport velocity following the application of 0.47 µM cafedrine/theodrenaline 20:1.
